# Management of a difficult airway for multispace infection of the floor of the mouth: A case report

**DOI:** 10.1097/MD.0000000000046405

**Published:** 2026-05-12

**Authors:** Bucheng Liao, Wuhao Liao, Zilin Wang, Shuang Yin

**Affiliations:** a Department of Anesthesiology, Peking University Shenzhen Hospital, Shenzhen, China; b Department of Anesthesiology, Shenzhen Hospital, Southern Medical University, Shenzhen, China.

**Keywords:** awake tracheal intubation, case report, difficult airway, multispace infection of the floor of the mouth

## Abstract

**Rationale:**

Multispace infections of the floor of the mouth are rapidly progressive, life-threatening conditions that often result in airway compromise due to anatomical distortion and trismus. Managing the airway in such cases is particularly challenging, especially when compounded by systemic metabolic derangements such as diabetic ketoacidosis. This case highlights a novel and safe approach to securing the airway in a critically ill patient when conventional methods are contraindicated.

**Patient concerns:**

A 27-year-old male with type 1 diabetes mellitus presented with diabetic ketoacidosis (pH 6.996, glucose 22.2 mmol/ L), severe trismus (interincisal distance <1.5 cm), maxillofacial swelling, cervical rigidity and dyspnea. Magnetic resonance imaging confirmed multispace infection with cervical abscess, mediastinal extension and pleural effusion.

**Diagnoses:**

Life-threatening multispace infection of the floor of the mouth complicated by diabetic ketoacidosis, cervical abscess, and imminent airway obstruction requiring emergency surgical drainage and tracheostomy.

**Interventions:**

Awake tracheal intubation (ATI) was performed under conscious sedation (dexmedetomidine and remifentanil) with spontaneous ventilation preserved. Sequential topical airway anesthesia (2% lidocaine) was administered via an epidural catheter secured to a fiberoptic bronchoscope. Following successful ATI, general anesthesia was induced for incision, drainage of abscesses, and tracheostomy.

**Outcomes:**

ATI was successfully completed without episodes of hypoxia, hemodynamic instability, or patient discomfort. Surgical incision and drainage of submental and sublingual abscesses, along with tracheostomy, were completed within 65 minutes under general anesthesia. Postoperatively, the patient regained spontaneous breathing on day 1, with a total of 76 mL of purulent drainage recorded. The patient was discharged on postoperative day 21 in stable clinical condition, with no signs of residual infection. No adverse events related to airway management were observed.

**Lessons:**

This is the first reported case of diabetic ketoacidosis with severe trismus and multispace infection managed by ATI using an epidural catheter fixed to a channel-less fiberoptic bronchoscope for sequential topical anesthesia. The technique offers an alternative when standard devices are impossible or contraindicated.

## 1. Introduction

Infection of multispace of the floor of the mouth, also referred to as cellulitis of the floor of the mouth, is a diffuse cellulitis that involves the bilateral submandibular, sublingual, and submental spaces. If not treated promptly, it can lead to rapid progression of maxillofacial infection and may result in severe complications. Despite aggressive antimicrobial therapy and surgical intervention, the mortality rate among these patients can still reach up to 40%.^[1]^ In a study on airway management for patients with maxillofacial infections,^[2]^ all 361 patients underwent surgical incision and drainage within 24 hours of admission. The case reported herein is extremely rare, presenting with diabetic ketoacidosis, limited neck mobility, severe trismus, and multispace infection of the floor of the mouth. In this scenario, we successfully managed the airway through awake tracheal intubation (ATI) using an epidural catheter for sequential topical anesthesia. This approach not only ensured airway patency but also minimized the risk of complications associated with invasive procedures. We describe the anesthetic strategy and discuss its potential application when standard airway topicalization is contraindicated or equipment is limited.

## 2. Case report

**Figure 1.**
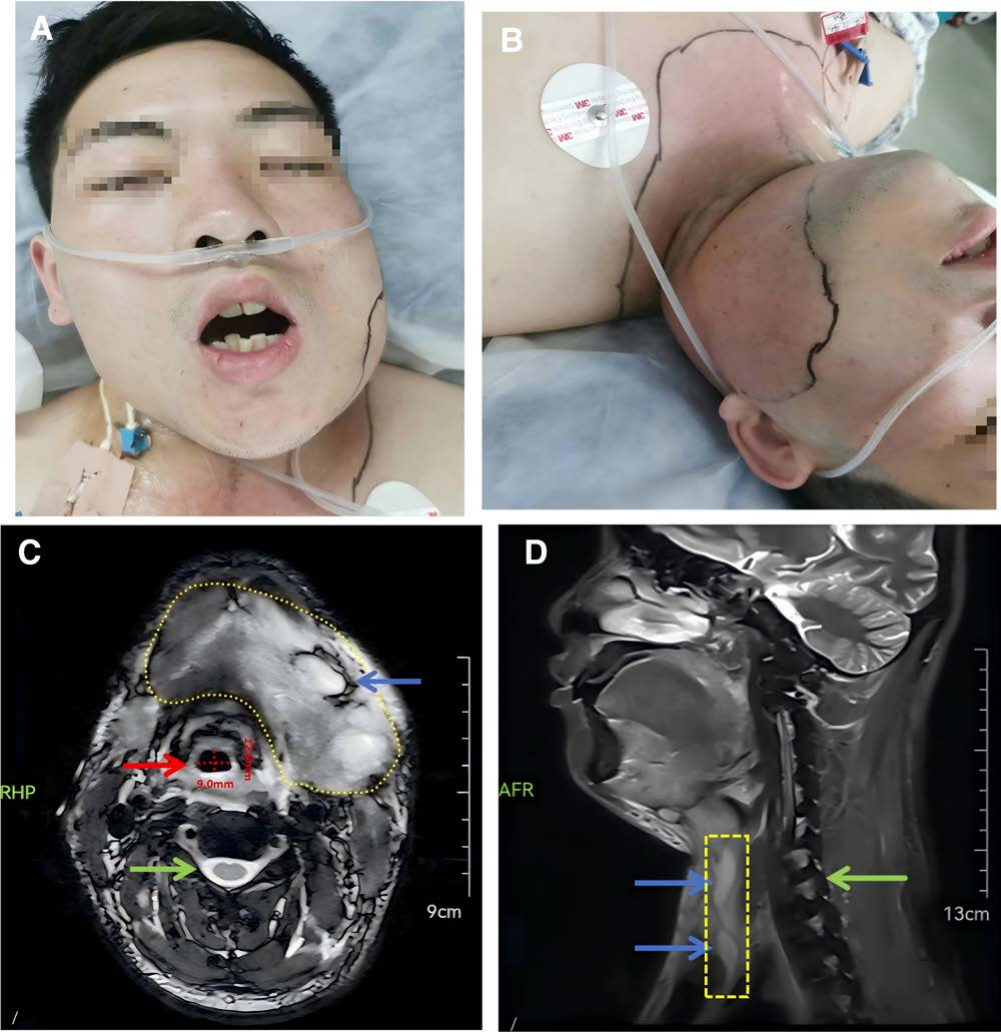
(A, B) Pre-anesthesia induction assessments reveal significant trismus and erythematous, edematous maxillofacial regions. (C, D) Magnetic resonance imaging (MRI) demonstrates abscess cavities (blue arrows), the surrounding swollen tissues (yellow areas), and the cervical vertebrae (green arrows) in both the axial (C) and sagittal (D) sections. The axial view (C) of the MRI reveals a compressed trachea (red arrow) with an anteroposterior diameter of 9.0 mm and a transverse diameter of 12.6 mm. MRI diagnosis: (1) Multiple abnormal signal shadows were observed in the left submandibular gland region, submandibular space, and adjacent to the thyroid gland. Subcutaneous exudation was noted in the left neck and submental area, suggestive of an infectious process. (2) There is evident swelling of the left lateral pterygoid muscle and masseter muscle.

A 27-year-old man was admitted with a 6-year history of polydipsia and polyuria, toothache for 3 days, and dyspnea for 9 hours. He had been diagnosed with diabetic ketoacidosis 6 years earlier and had since used insulin as part. Three weeks before admission he self-discontinued insulin. Left-sided toothache began 3 days prior to admission and was partially relieved by amoxicillin. Nine hours before presentation he developed dyspnea, Kussmaul respiration, dry mouth and vomiting. Laboratory values: pH 6.996, lactate 2.7 mmol/L, glucose (GLU) 22.2 mmol/L, urine ketones 3+. After resuscitation with antibiotics, fluids and acidosis correction in the intensive care unit (ICU), he reported mild dyspnea, intermittent chest tightness and marked left facial swelling. Magnetic resonance imaging (MRI) of the maxillofacial region is shown in Figure 1. Diagnosis: Type 1 diabetes mellitus with diabetic ketoacidosis; Maxillofacial space infection; Deep cervical abscess; Mediastinal abscess. Under general anesthesia, the following procedures are planned: Incision and drainage of the submental and sublingual abscesses; Tracheostomy.

**Figure 2.**
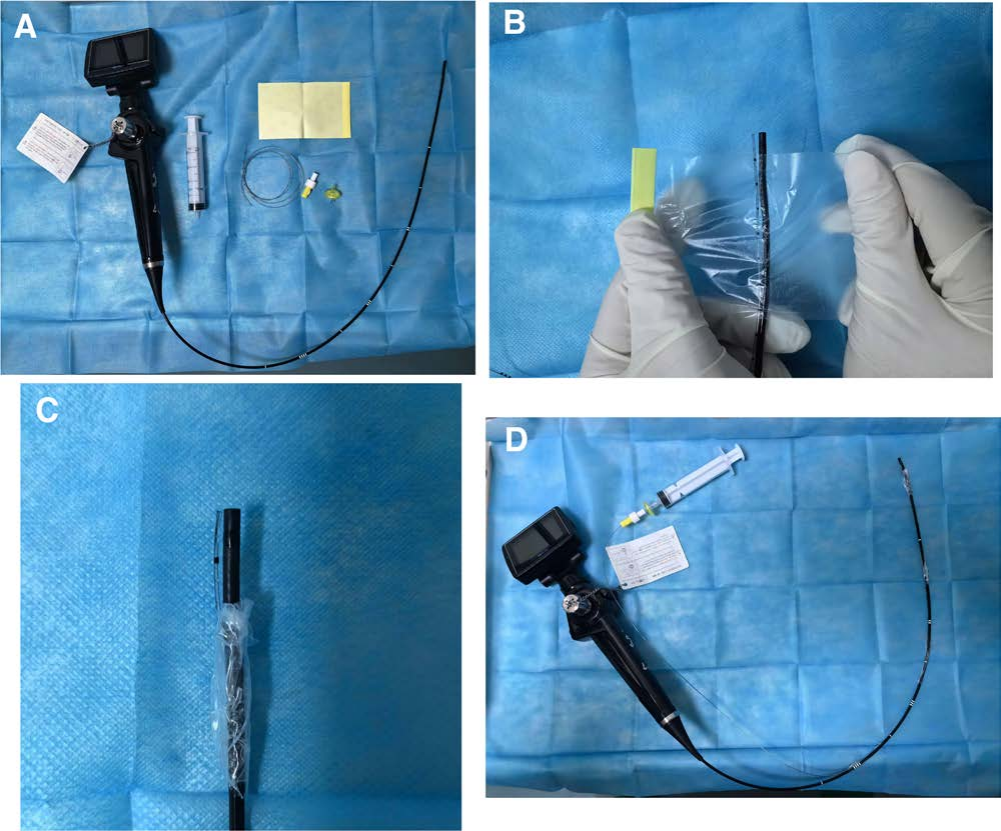
(A) Prepare the necessary equipment and materials for the procedure. (B, C) Secure the catheter to the bronchoscope every 5 cm with transparent tape to prevent displacement. (D) Image following the securement of the bronchoscope and epidural catheter.

Preoperative management focused on aggressive infection control, normoglycaemia and correction of any internal milieu derangements. Upon arrival in the operating theater, a comprehensive assessment was performed: the patient exhibited pronounced maxillofacial swelling with severe trismus (interincisal distance <1.5 cm) and restricted cervical motion (Fig. 1). Noninvasive blood pressure, pulse oximetry (SpO_2_) and 5-lead electrocardiography were instituted without delay. GLU was measured at 7 mmol/L. Peripheral intravenous access was established, and a right radial artery cannulation was performed under local anesthesia for continuous arterial blood pressure monitoring. The patient was informed about the planned nasal tracheal intubation under spontaneous breathing and conscious sedation, and his understanding and cooperation were obtained. The following steps were taken during the procedure: continuous oxygen inhalation (oxygen flow rate: 5 L/min), intravenous infusion of dexmedetomidine and remifentanil by pump, with dosages of 0.8 mcg/kg (for 10 minutes, total 42 mcg) and 0.07 mcg/kg/min (ATI), respectively. Concurrently, we used a fiberoptic bronchoscope to guide the epidural catheter for infiltration anesthesia. The specific operation steps are as follows: First, prepare all necessary equipment and consumables, including a fiberoptic bronchoscope, a transparent dressing, a 20-mL syringe, an epidural catheter and connector (Fig. 2A). A fiberoptic bronchoscope (outer diameter 3.5 mm) without a working channel was used for nasal intubation. Because the scope lacks a drug-delivery port, an epidural catheter was secured alongside its shaft with transparent dressing to allow sequential topical administration of 2% lidocaine (Fig. 2B and C). The catheter was secured to the bronchoscope every 5 cm with transparent tape. Finally, at the proximal end of the epidural catheter, use transparent tape for the final fixation to ensure a firm and reliable connection between the catheter and the bronchoscope. After completing the fixation, connect the syringe to the proximal end of the epidural catheter (Fig. 2D).

**Figure 3.**
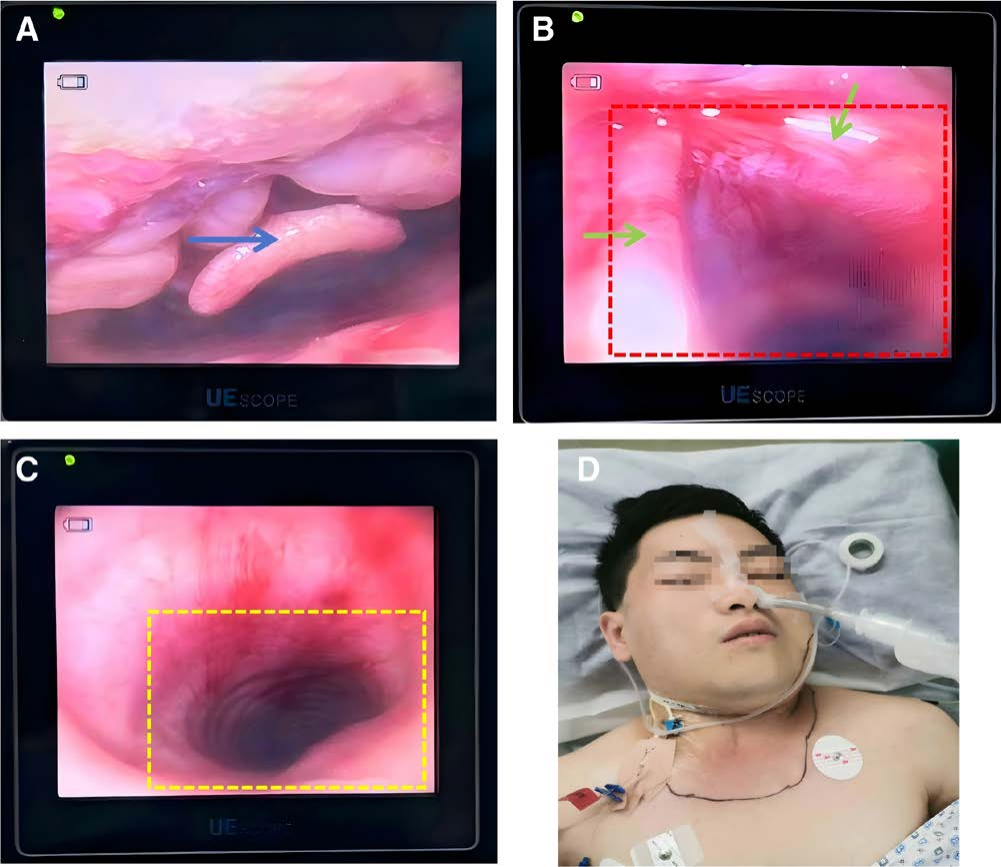
(A–C) Fiberoptic bronchoscopy-guided tracheal intubation reveals the conditions of the patient’s epiglottis (blue arrows), bilateral vocal cords (green arrows), glottis (red area), and main trachea (yellow area). (D) Post-procedure images following successful awake tracheal intubation (ATI). The distance from the tip of the nose to the tip of the tracheal tube was measured to be 26 cm.

**Figure 4.**
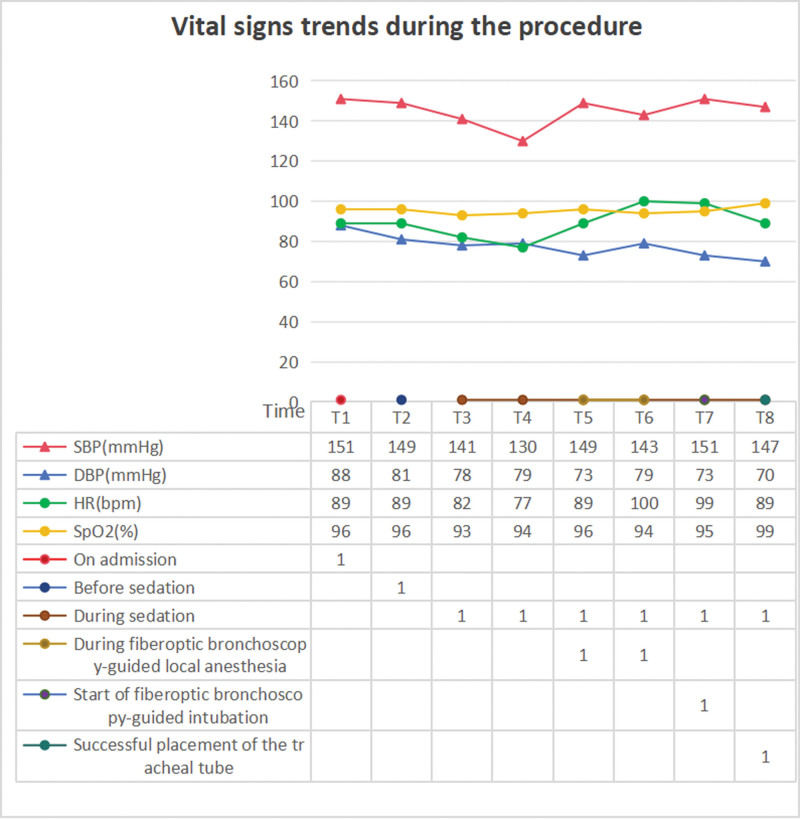
Vital signs trends during the procedure. SBP = systolic blood pressure, DBP = diastolic blood pressure, NIBP = noninvasive blood pressure, HR = heart rate, SpO_2_ = saturation of peripheral oxygen, T1 = on admission, T2 = before sedation, T3–T8 = during sedation, T5–T6 = during fiberoptic bronchoscopy-guided local anesthesia, T7 = start of fiberoptic bronchoscopy-guided intubation, T8 = successful placement of the tracheal tube.

Ten minutes after the infusion of dexmedetomidine and remifentanil, the patient was in a sedated state, with spontaneous breathing at 16 breaths per minute and saturation of peripheral oxygen at 96%. We used a 20 mL syringe to draw 2% lidocaine (total 150 mg) for local anesthesia and gradually sprayed the local anesthetic through the epidural catheter into the nasal cavity, nasopharynx, oropharynx, glottis and main trachea, approximately 20 to 40 mg for each area, achieving effective infiltration anesthesia. After the completion of airway surface anesthesia, a sterile endotracheal tube was placed over the fiberoptic bronchoscope. Under direct visualization, the fiberoptic bronchoscope was inserted through the nasal cavity and advanced in a neutral position. After entering the pharynx and locating the epiglottis, the tip of the bronchoscope was passed beneath the epiglottis and the glottis was visualized. At this point, the bronchoscope was adjusted to enter the trachea until it reached 4 to 6 cm above the carina. The endotracheal tube was then advanced along the bronchoscope into the trachea. After confirming correct placement in the trachea, the tube was secured (Fig. 3). The vital signs of the patient from entering the operating room to the completion of awake intubation were presented in Figure 4. General anesthesia was induced with 150 mg of propofol, 50 mg of rocuronium, and 10 mcg of sufentanil. During the operation, anesthesia was maintained with 2% sevoflurane and remifentanil at a rate of 0.1 mcg/kg/min. The incision and drainage of the maxillofacial abscess and tracheotomy were successfully completed within 1 hour and 5 minutes. Prior to exiting the operating room, the patient’s GLU was recorded at 9.9 mmol/L. The intraoperative blood loss was minimal at 5 mL, and a total fluid infusion volume of 1250 mL was administered. Following the operation, the patient was safely transferred to the ICU under controlled mechanical ventilation.

The patient resumed spontaneous breathing on postoperative day 1, with 76 mL of yellow exudate drained. Postoperatively, ceftriaxone sodium and linezolid were administered for antimicrobial therapy, while the patient’s blood glucose and electrolyte levels were closely monitored and managed. After regaining full consciousness and return to spontaneous breathing, the patient was asked about his recall of the awake intubation period. He reported satisfaction with the airway management approach and denied any memory of discomfort during the procedure. On postoperative day 11, the patient was transferred to a general ward for continued wound care of the neck incision and infection prophylaxis. The patient was ultimately discharged in stable condition on postoperative day 21. No adverse or unanticipated events were observed during the procedure or within the postoperative hospital stay. The chronological sequence of major clinical events was summarized in Table 1.

**Table 1:** Timeline of major clinical events.

Time point	Event
Year ‐6	Diagnosis of type 1 diabetes mellitus with DKA
Day ‐3	Onset of toothache
Day 0	Emergency admission, ICU transfer, MRI
Day 1	ATI and surgery
Day 2	Spontaneous breathing resumed
Day 11	Transfer to general ward
Day 21	Discharge

ATI = awake tracheal intubation, Day 0 = day of emergency admission, DKA = diabetic ketoacidosis, ICU = intensive care unit, MRI = magnetic resonance imaging, Year ‐6 = 6 years before the index admission.

## 3. Discussion

The causes of multiple space infections in the floor of the mouth primarily include odontogenic origins (such as periodontitis, pericoronitis, and complications from dental procedures), adenogenic origins (such as tonsillitis), hematogenous spread, iatrogenic factors, and trauma to the maxillofacial region.^[3]^ The most common and serious life-threatening complication of multispace infection at the floor of the mouth is airway obstruction. The current patient has exhibited signs of respiratory distress. Prior research indicates that the presence of coughing, wheezing, and respiratory difficulty should be treated with utmost urgency. Therefore, it is strongly recommended that the patient be promptly admitted to the ICU and that an artificial airway be established without delay to reduce mortality.^[4,5]^ Given that the infection had spread to the neck and the patient was experiencing respiratory distress, our center promptly performed abscess incision and drainage, along with tracheotomy, to ensure airway patency and safeguard the patient’s life.

Prior to surgery, the patient was admitted to the ICU for management of electrolyte imbalances and other systemic issues. Concurrently, preoperative blood glucose levels were optimized to 7 mmol/ L. Consequently, the most urgent and challenging concern during anesthesia was the management of a difficult airway.

Initially, the surgical team considered performing an awake tracheostomy under local anesthesia. However, this plan was abandoned due to the anterior extension of the abscess, which posed a risk of rupture and bacterial dissemination from needle traversal through infected tissue. Given the patient’s severe trismus, both video-laryngoscopy-guided tracheal intubation and supraglottic airway device insertion were deemed infeasible. To ensure airway patency during surgery, we opted for fiberoptic bronchoscopy-guided tracheal intubation while preserving spontaneous breathing. At this point, adequate analgesia became essential. Bilateral superior laryngeal nerve blocks and other percutaneous puncture techniques were contraindicated due to the anterior neck abscess, as these procedures could facilitate infection tracking along the needle tract. Consequently, fiberoptic bronchoscopy-guided ATI combined with sequential topical anesthesia via an epidural catheter emerged as the least invasive and safest option, maintaining spontaneous breathing until successful intubation. In cases where a fiberoptic bronchoscope lacks a working channel, the “spray as you go” technique using an epidural catheter for sequential lidocaine administration is an effective method for achieving adequate airway anesthesia.^[6]^ This decision aligns with the Spanish guideline for difficult airway management,^[7]^ which recommends that “when tracheal intubation is deemed highly difficult or impossible, awake airway management techniques should be performed.” This approach ensures patient safety by preserving spontaneous ventilation and intrinsic airway tone until successful tracheal intubation, thereby mitigating the risks associated with managing difficult airways in anesthetized patients.^[8–10]^ Furthermore, the Spanish guideline for difficult airway management^[7]^ emphasize that ATI should be prioritized when composite predictors of anatomical, physiological, and contextual difficulty are present. In our patient, the coexistence of severe trismus, restricted neck mobility, metabolic instability, and anterior neck infection fulfilled these criteria, making ATI the most appropriate first-line approach. Moreover, the use of cognitive aids—such as pre-procedure checklists and structured airway carts—can support decision-making and reduce error in high-stress situations, although they were not formally used in this case.^[7]^

Additionally, due to the rapid completion of awake intubation and the absence of copious secretions, antisialogogues such as glycopyrrolate or atropine were not administered (Fig. 3). Although the Difficult Airway Society guidelines for ATI in adults^[11]^ state that antisialogogues are not mandatory, their use should be considered when excessive secretions are anticipated to improve visualization.

Given the potential for increased stress upon entry of the fiberoptic bronchoscope into the airway, we took a proactive approach to sedation and analgesia. We administered 2% lidocaine via an epidural catheter from the nasal cavity to the airway in a stepwise manner. To mitigate the anticipated stress and ensure patient comfort, we co-administered dexmedetomidine and remifentanil. The sedation regimen for this case was guided by the research of Yi Zhou et al,^[12]^ which demonstrated the combined use of dexmedetomidine and remifentanil during fiberoptic bronchoscopy to achieve a balance between sedation and spontaneous breathing.

In this case, dexmedetomidine and remifentanil were given by conventional weight-based continuous infusion rather than the target-controlled infusion (TCI) reported by Yi Zhou et al.^[12]^ This deviation was necessitated by institutional equipment constraints: the syringe pumps available in our operating theaters lack embedded TCI firmware for remifentanil. Both strategies share an identical therapeutic endpoint—adequate sedation while preserving spontaneous ventilation. At the fixed infusion rate administered (remifentanil: 0.07 mcg/kg/min), the resultant pharmacodynamic effect mirrored the TCI-derived EC95 of 2.710 ng/mL described by Yi Zhou et al.^[12]^ Although the patient maintained spontaneous breathing under adequate sedation while tolerating the reflex responses associated with fiberoptic bronchoscopy, we emphasize that the safety and efficacy of constant-rate remifentanil for awake intubation require validation in larger cohorts. Continuous monitoring of respiratory rate and oxygenation throughout the procedure confirmed stable spontaneous ventilation during the entire awake intubation period. During the entire ATI process, the opioid receptor antagonist naloxone should be prepared to ensure timely treatment in case of severe respiratory depression related to remifentanil. During nasal tracheal intubation, the patient experienced only occasional coughing and did not report any other discomfort. This case confirms that, in addition to bilateral superior laryngeal nerve block and cricothyroid membrane puncture injection of local anesthetic, the sequential administration of local anesthetic via a epidural catheter is another viable method for airway surface anesthesia.

Our technique of securing an epidural catheter to the fiberscope has several limitations: first, it may prolong preparation time; second, epidural catheter topicalization is suitable only for bronchoscopes without a working channel, and the standard intraluminal route should be used when a channel is available; third, adequate hands-on training is required before clinical application, and the safety and success rate of this method need validation in large-scale studies.

Additionally, in this case, the anteroposterior and transverse diameters of the trachea at its narrowest point were measured to be approximately 9.0 and 12.6 mm, respectively. Consequently, a tracheal tube with an inner diameter of 6.5 mm (outer diameter approximately 8.9 mm) was selected. During the administration of local anesthetics via the epidural catheter guided by fiberoptic bronchoscopy, we utilized a small-diameter bronchoscope (outer diameter 3.5 mm) to minimize patient discomfort. However, this particular model lacks a drug-delivery channel. Consequently, we fabricated an epidural catheter for drug administration, which represents one of the key innovations in this case.

In conclusion, when anesthesiologists encounter patients with multispace infections of the floor of the mouth, they must carefully assess the patient’s overall condition, with particular emphasis on airway management. If the patient exhibits signs of a potentially difficult airway, such as trismus or limited neck mobility, it is advisable to perform tracheal intubation while maintaining spontaneous ventilation and consider performing a tracheotomy intraoperatively if necessary. It should be emphasized that this report describes a single case; consequently, these findings require validation through larger studies before they can be generalized. The technique outlined—using an epidural catheter attached to a fiberoptic bronchoscope to provide airway anesthesia—requires meticulous preparation and may not be feasible in emergency settings without adequate training or equipment.

## Author contributions

**Conceptualization**: Bucheng Liao.

**Data curation**: Zilin Wang.

**Investigation**: Bucheng Liao, Zilin Wang, Shuang Yin.

**Supervision**: Wuhao Liao, Shuang Yin.

**Writing – original draft**: Bucheng Liao.

**Writing – review & editing**: Shuang Yin.
